# Pharmacological therapy of metabolic dysfunction-associated steatotic liver disease-driven hepatocellular carcinoma

**DOI:** 10.3389/fphar.2023.1336216

**Published:** 2024-01-19

**Authors:** Yumin Wang, Joshua S. Fleishman, Tongda Li, Yulin Li, Zhao Ren, Jichao Chen, Mingchao Ding

**Affiliations:** ^1^ Department of Respiratory and Critical Care Medicine, Aerospace Center Hospital, Peking University Aerospace School of Clinical Medicine, Beijing, China; ^2^ Department of Pharmaceutical Sciences, College of Pharmacy and Health Sciences, St. John’s University, Queens, NY, United States; ^3^ Department of Traditional Chinese Medicine, Beijing Geriatric Hospital, Beijing, China; ^4^ Department of Pharmacy, Aerospace Center Hospital, Peking University Aerospace School of Clinical Medicine, Beijing, China; ^5^ Department of Peripheral Vascular Intervention, Aerospace Center Hospital, Peking University Aerospace School of Clinical Medicine, Beijing, China

**Keywords:** hepatocellular carcinoma, non-alcoholic fatty liver disease, metabolic dysfunction-associated steatotic liver disease, natural products, treatment

## Abstract

In light of a global rise in the number of patients with type 2 diabetes mellitus (T2DM) and obesity, non-alcoholic fatty liver disease (NAFLD), now known as metabolic dysfunction-associated fatty liver disease (MAFLD) or metabolic dysfunction-associated steatotic liver disease (MASLD), has become the leading cause of hepatocellular carcinoma (HCC), with the annual occurrence of MASLD-driven HCC expected to increase by 45%–130% by 2030. Although MASLD has become a serious major public health threat globally, the exact molecular mechanisms mediating MASLD-driven HCC remain an open problem, necessitating future investigation. Meanwhile, emerging studies are focusing on the utility of bioactive compounds to halt the progression of MASLD to MASLD-driven HCC. In this review, we first briefly review the recent progress of the possible mechanisms of pathogenesis and progression for MASLD-driven HCC. We then discuss the application of bioactive compounds to mitigate MASLD-driven HCC through different modulatory mechanisms encompassing anti-inflammatory, lipid metabolic, and gut microbial pathways, providing valuable information for future treatment and prevention of MASLD-driven HCC. Nonetheless, clinical research exploring the effectiveness of herbal medicines in the treatment of MASLD-driven HCC is still warranted.

## Introduction

As the most common type of primary liver cancer, hepatocellular carcinoma (HCC) represents the fifth most common cancer worldwide (Akinyemiju et al., 2017). Currently, HCC is on the path to globally becoming the second most common cause of cancer-related death. Historically, cirrhosis induced by chronic infections (such as hepatitis B/C virus) and alcoholic hepatotoxicity are the two major causes of HCC (Villanueva, 2019). However, the incidence of chronic infection-associated HCC is decreasing with the development of anti-HCV drugs and anti-HBV vaccines (Akinyemiju et al., 2017). Accumulating evidence suggests that HCC-associated mortality is increasing steadily, indicating that other risk factors besides alcohol and viral hepatitis drive HCC pathogenesis.

**FIGURE 1:**
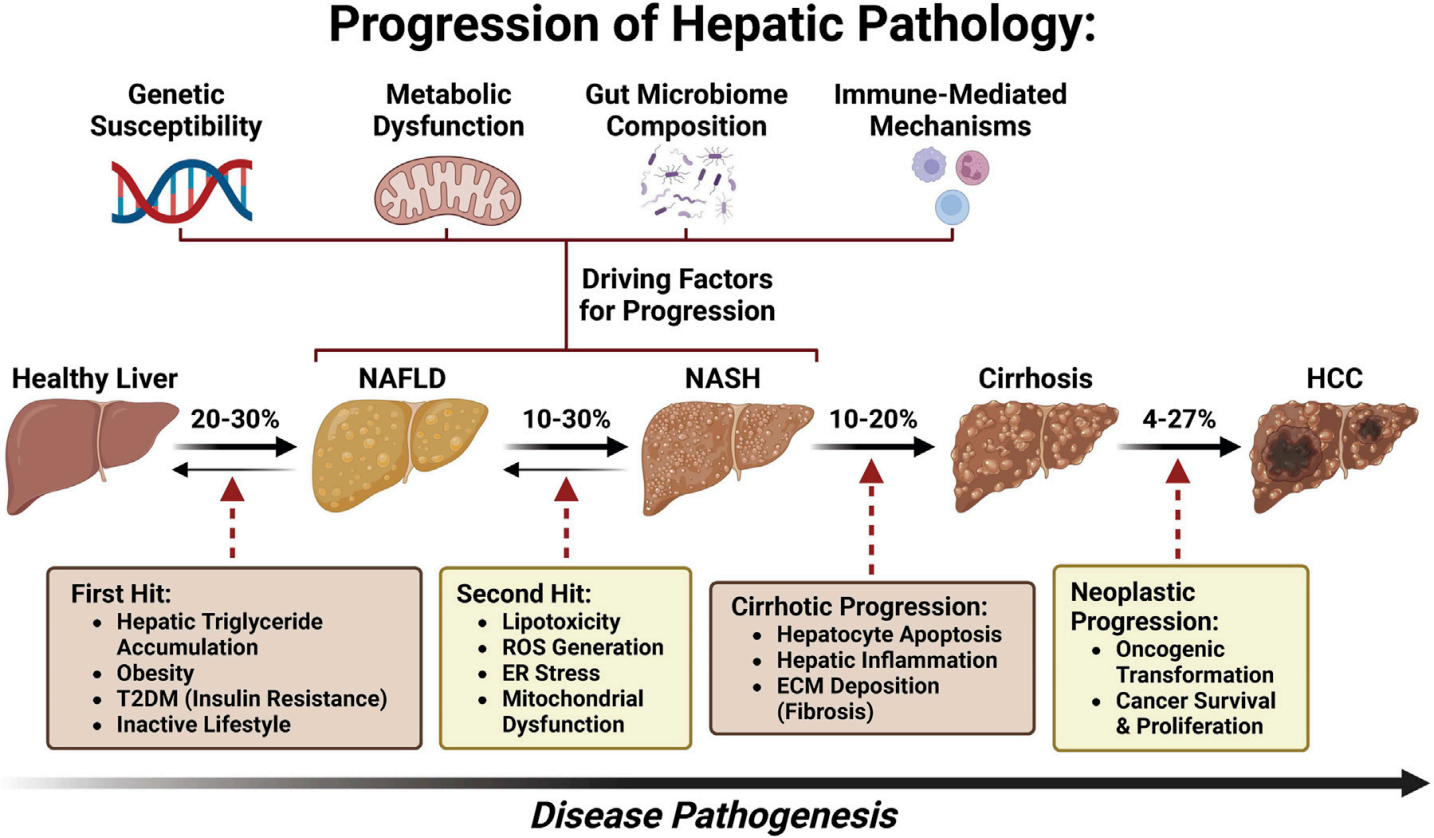
Pathogenesis spectrum of MASLD/MASH–cirrhosis–HCC.

With a global rise in the number of patients with type 2 diabetes mellitus (T2DM) and obesity, non-alcoholic fatty liver disease (NAFLD), now named metabolic dysfunction-associated fatty liver disease (MAFLD) (Eslam et al., 2020a; Eslam et al., 2020b; Lim et al., 2021; Pirola and Sookoian, 2022; Sangro et al., 2023) or metabolic dysfunction-associated steatotic liver disease (MASLD) (Yang et al., 2023; Rinella et al., 2023a; Rinella et al., 2023b; Rinella et al., 2023c; Noureddin et al., 2023; He et al., 2023; Chan et al., 2023; Hagström et al., 2023), has been postulated to become the leading etiology of HCC, leading to MASLD-associated hepatocellular carcinoma (MASLD-HCC) (Younossi et al., 2018; Zhou et al., 2019; Sarin et al., 2020; Talamantes et al., 2023). The prevalence of MASLD-HCC is increasing in most countries worldwide and presents a major healthcare burden (Koh et al., 2024). However, the underlying molecular mechanism of MASLD progression to HCC remains largely unknown (Yu et al., 2020; Di Maira et al., 2022). As a disease continuum progressing from simple steatosis to non-alcoholic steatohepatitis (NASH), now named as metabolic dysfunction-associated steatohepatitis (MASH) and fibrosis, MASLD affects 25% of adults and is one of the most common chronic liver diseases globally (Bence and Birnbaum, 2021; Powell et al., 2021; Kalligeros et al., 2023). Approximately 27% of cirrhosis induced by MASH could progress to MASLD-HCC (Ioannou, 2021) (Figure 1). Although several drugs are being tested for MASLD/MASH treatment, no curative agents are effective against MASLD-HCC (Wong, 2018). Therefore, there is an unmet need for the discovery of novel and safe drugs for MASLD-HCC. Meanwhile, emerging bioactive compounds have been shown to halt the progression of MASLD to MASLD-driven HCC (Khairnar et al., 2023).

This article aims to assess the recent advancements in pharmacological therapy against MASLD-HCC. In this review, we first briefly review the possible main mechanisms of the progression and pathogenesis of MASLD-HCC. Then, we overview the application of bioactive compounds in mitigating MASLD-HCC. We categorize drugs that treat MASLD-HCC by their mechanisms (anti-inflammatory, lipid metabolism, and gut microbiota), providing valuable information for future treatment and prevention of MASLD-HCC.

## Distinctive features of MASLD-HCC

Patient demographics have revealed that patients with MASLD and HCC are mainly White, male, and older than patients with HCC from other origins (Degasperi and Colombo, 2016). A prospective multicenter study in Italy indicates that compared to patients with hepatitis C-related tumors, 54% of MASLD-HCC patients had no evidence of cirrhosis histologically or clinically, were younger, and were less frequently diagnosed during surveillance (Piscaglia et al., 2016). MASLD-HCC tumors tended to be larger and fell less frequently within the Milan criteria or in BCLC stage 0. Compared to HCV-related HCC, MASLD-HCC manifested an infiltrative pattern (Piscaglia et al., 2016). This observation was corroborated by a retrospective cohort HCC study at the Veterans Affairs Hospitals in the United States, in which patients with hepatitis C HCC were younger than those with MASLD-HCC (Mittal et al., 2015). HCC in veterans with MASLD was distinctively characterized by diabetes and dysmetabolism, including peripheral arteriopathy, myocardial infarction, and congestive heart failure. Owing to the burdens of age and comorbidities, patients in the hepatitis C group had a higher likelihood of curative treatment than those with MASLD-HCC (22% VS 11%) (Mittal et al., 2015). A more recent study provided compelling evidence that HCC may also develop in MASLD patients with persistently normal values of transaminase activity (Natarajan et al., 2020).

It has been shown in both population-based and large cohort studies that MASLD patients have an increased risk for metabolic syndrome and diabetes complication-associated cardiovascular comorbidities compared with patients with HCC without MASLD. The median age was 73 years and 66 years in patients with MASLD and hepatitis C, respectively, as per the SEER Registry (Younossi et al., 2015). White patients accounted for 57% (1,560/2,536) of 2,536 patients with hepatitis C and 76% (532/701) of 701 cases with MASLD (Younossi et al., 2015). These results aligned well with the findings of the cohort study of 1,500 American veterans with HCC, where it was reported that the prevalence of arterial hypertension (95% vs 70%) and diabetes (89% vs 33%) was higher in the MASLD patient group than in patients with hepatitis C (Mittal et al., 2015). MASLD patients had significantly higher rates of cardiovascular disease and peripheral vascular disease. Mittal’s study uncovered that a poor prognosis in patients with MASLD results from the burden of comorbidities, leading to limited access to more radical treatments, such as hepatic resection or transplantation (Mittal et al., 2015). A recent retrospective study replicated these findings, which compared the clinicopathological characteristics and prognosis of patients undergoing surgical resection in MASLD/MASH-associated HCC and other HCC etiologies (Pal Chaudhary et al., 2023). A total of 110 HCC patients had MASLD/MASH, and 150 patients had other etiologies. The median age at diagnosis was lower in the other etiology cohort than in the MASLD/MASH-HCC cohort, with a decreased number of female patients (Pal Chaudhary et al., 2023). The diameter of tumors induced by MASLD/MASH was more commonly >5 cm. There were no significant differences in rates of lymphovascular or perineural invasion, histologic grade, or serum AFP levels. There was a lower rate of background liver fibrosis, lower aspartate transaminase, lower alanine aminotransferase, and higher platelet counts in the MASLD/MASH cohort (Pal Chaudhary et al., 2023). These findings suggest that patients with MASLD/MASH-HCC more commonly presented with larger HCC tumors and lacked liver fibrosis than those with HCC due to other etiologies.

## Pathogenesis of MASLD-driven HCC

**FIGURE 2:**
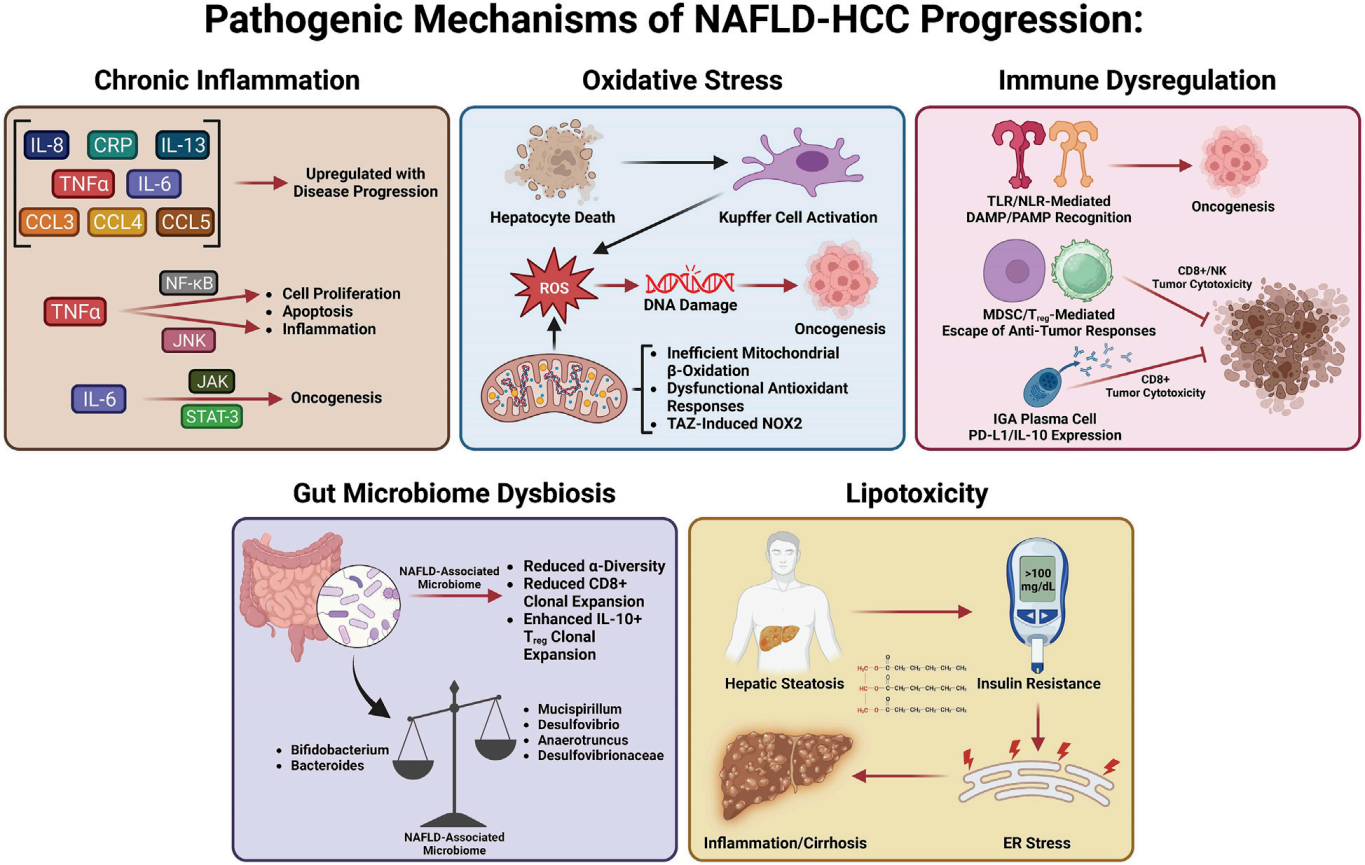
Pathogenic mechanisms of MASLD-HCC progression.

Many studies have investigated the specific mechanisms of transition from MASLD/MASH to HCC. It is thought that this process involves multiple factors including oxidative stress, lipotoxicity, gut dysbiosis, metabolic imbalances, chronic injury, and hypoxia, which, in turn, stimulate chronic inflammation, tissue scarring, and HCC pathogenesis (Cannito et al., 2023). In this section, we will summarize the current knowledge about these factors and how they may be involved in the transition from MASLD/MASH to HCC (Figure 2).

### Chronic inflammation in MASLD-HCC

Many variables play a role in the MASLD to MASH transition, including metabolic dysfunction, oxidative stress, gut dysbiosis, lipotoxicity, and hepatocellular necrosis, all of which induce chronic inflammation, leading to perpetual tissue injury, parenchymal cell regeneration, mutagenesis, and HCC tumorigenesis. Hepatic oxidative stress, inflammation, and insulin resistance (IR) are important features and hallmarks of MASLD-HCC (Talamantes et al., 2023). Enhanced inflammation and IR are found in patients with HCC, evidenced by elevated levels of pro-inflammatory cytokines such as interleukin-6 (IL-6), tumor necrosis factor-α (TNF-α), and C-reactive protein (Aleksandrova et al., 2014; Duan et al., 2022). Both obesity and IR systemically are responsible for HCC tumorigenesis as they induce inflammation and activate oncogenic pathways (Margini and Dufour, 2016). The chronic low-grade inflammation that has been linked to HCC development is a distinctive feature of MASLD (Talamantes et al., 2023). Hepatocellular injury due to persistent stimulation of innate and adaptive immune pathways, gut dysbiosis, and low-grade chronic systemic inflammation in MASLD (Cannito et al., 2023) all contribute to the pathogenesis of MASLD-HCC (Marengo et al., 2016; Polyzos et al., 2023; Talamantes et al., 2023). The liver is chronically exposed to TNF-α and IL-6, the two major obesity-associated adipose-derived pro-inflammatory cytokines (Avgerinos et al., 2019; Potoupni et al., 2021). Cytokines facilitate inflammatory and immune tolerance in the liver microenvironment. Chemokines have also been associated with the pathogenesis of MASLD-HCC. It was found that MASLD-HCC patients have higher plasma levels of IL-8, IL-13, chemokine (C–C motif) ligand (CCL-3), CCL-4, and CCL-5, which are correlated with activated circulating monocytes (Ponziani et al., 2019). Several signaling pathways linked to inflammation, steatosis, and oncogenes can be activated by IL-6 and TNF-α in hepatocytes. Upregulation of TNF-α and IL-6 promoted hepatic steatosis and inflammation in a mouse obesity model (Park et al., 2010). Of note, Janus kinase/signal transducer and activator of transcription 3 (JAK/STAT-3) and c-Jun N-terminal kinase/nuclear factor kappa B (JNK/NF-κB) pathways are commonly activated in HCC (Llovet et al., 2021). TNF-α activates the NF-κB and JNK pathways, inducing the transcription of genes involved in hepatic cell proliferation, inflammation, and apoptosis (Yu et al., 2013). IL-6 activates the JAK/STAT-3 pathway, which is linked to cell differentiation and cell growth (Grohmann et al., 2018). Furthermore, oncogenic STAT3 activity associated with HCC pathogenesis is induced by both the inflammatory response and steatosis (Park et al., 2010). The detailed role of intracellular mitogenic, anti-apoptotic, and other signaling pathways involved in the pathogenesis of MASLD-HCC has been recently reviewed [see (Polyzos et al., 2023; Talamantes et al., 2023) for a recent review].

### Oxidative stress in MASLD-HCC

Accumulating evidence reveals that the role of oxidative stress in the pathogenesis of MASLD-HCC is well-elucidated and established, with precise mechanistic exploration recently published (Brahma et al., 2021; Gabbia et al., 2021). Excessive hepatic lipid exposure triggers oxidative stress-dependent cell damage through different mechanisms (Bessone et al., 2019). Oxidative stress and ensuing hepatocyte death substantially promote cell proliferation and activate hepatic resident Kupffer cells simultaneously, which secrete chemokines and cytokines to recruit other immune cells, thereby intensifying inflammation and further facilitating the production of reactive oxygen species (ROS) (Wen et al., 2021). At the same time, chronic oxidative stress promotes genomic DNA mutations and upregulates HCC development-related genes (Wen et al., 2021). Increased lipid accumulation can generate pathogenic drivers of carcinogenesis in hepatocytes, in particular oxidative DNA damage (Tanaka et al., 2013; Masarone et al., 2018), which has been found higher in the hepatocytes of MASH-HCC patients than in the hepatocytes of MASH patients without HCC(Tanaka et al., 2013). Inadequate mitochondrial respiratory chain activity and elevated mitochondrial fatty acid β-oxidation (FAO) lead to production of ROS in hepatocytes that can damage DNA in MASLD (Begriche et al., 2013). Dysfunction of antioxidant cellular mechanisms can result in an elevation in fatty acid (FA) metabolism-associated pathways, which causes fatty acid accumulation, steatosis, and metabolic stress (Masarone et al., 2018). Enhanced FA accumulation leads to *de novo* lipogenesis (DNL) and FAO, which promotes ROS generation (Perla et al., 2017). Hepatic steatosis functions as a precursor for ROS generation and can promote STAT-1 and STAT-3 activity through oxidizing constituently active phosphatases, driving MASH, fibrosis, and HCC pathogenesis (Grohmann et al., 2018). Since increased oxidative stress and ROS have been linked to the transition from MASLD to HCC, emerging studies have focused on uncovering the role of NADPH oxidases (NOXs) in MASLD-HCC. Wang et al. (2022) have shown that the transducer of the Hippo pathway, transcriptional co-activator with PDZ binding motif (TAZ), contributes to MASH-associated HCC in pre-tumor MASH hepatocytes via induction of Cybb and NOX2-mediated DNA damage. This interesting study provided compelling evidence that TAZ plays a role in the progression of MASH to HCC and suggested a future therapeutic option for treating MASH-HCC by targeting MASH through TAZ. Wang et al. (2022) provided a new research paradigm to assess the molecular mechanisms related to MASLD/MASH-HCC, observing pathogenesis by modulating TAZ-mediated NOX2-derived oxidative damage to the DNA of hepatocytes. The author suggested that TAZ-mediated therapies may be a potential avenue for MASH-HCC treatment.

### Immune dysregulation in MASLD-HCC

The role of the immune response in the pathogenesis of MASLD-HCC has been comprehensively revealed and summarized elsewhere [see (Anstee et al., 2019; Hirsova et al., 2021; Zhang and Yang, 2021; Riaz et al., 2022; Yahoo et al., 2023) for a recent review]. In short, chronic inflammation induced by pathogen-associated molecular patterns (PAMPs) and damage-associated molecular patterns (DAMPs) serves as a significant driver of hepatocarcinogenesis (Park et al., 2010; Arrese et al., 2016). Vital immunosuppression by T regulatory cells (Tregs) and myeloid-derived suppressor cells (MDSCs) on cytotoxic CD8^+^ T cells and natural killer (NK) cells mediates immune escape from antitumor responses. Targeting the recruitment of MDSCs and Tregs into the liver is a potentially druggable step in the pathogenesis of MASH-HCC, especially after observing that platelet-derived platelet glycoprotein Ibα (GPIbα) proved critical for the development of MASH-HCC (Malehmir et al., 2019). The role of adaptive immunity in the pathogenesis of MASLD-HCC has also been revealed, showing that IgA^+^ plasma cells accumulate in MASH fibrosis and lead to MASLD-HCC by inhibiting CD8^+^ T cells via IL-10 and programmed cell death ligand 1 (PD-L1) expression (Shalapour et al., 2017). These immunosuppressive IgA^+^ plasma cells may play a key role in the gut microbiome–liver axis, contributing to HCC carcinogenesis by providing mucosal IgA exposure.

### Gut microbiome dysbiosis in MASLD-HCC

Recent studies have shown that gut microbiota dysbiosis contributes to MASLD-HCC formation, even in the absence of cirrhosis (Xie et al., 2016; Sydor et al., 2020; Yahoo et al., 2023). Emerging studies demonstrate that the intestinal microbiota play a role in stimulating and maintaining liver inflammation, which becomes more pro-inflammatory as the disease progresses toward HCC (Xie et al., 2016; Sydor et al., 2020). This initial animal study was corroborated by a clinical study, which reported a decreased abundance of *Bifidobacterium* and an elevated abundance of Ruminococcaceae and *Bacteroides* in MASLD-HCC patients compared to patients with cirrhosis, which did not progress to MASLD-HCC (Ponziani et al., 2019). Decreased α-diversity (a measure of microbiome diversity applicable to a single sample) was found in patients with MASLD-HCC (Ponziani et al., 2019). Later work elaborated on this finding, demonstrating that a decreased α-diversity and the Chao-1 richness index were found in patients with MASLD-HCC (Behary et al., 2021). In addition, there is a relationship between the gut microbiota and several inflammatory cytokines such as higher levels of IL-8 and CCL-3 in patients with MASLD-HCC (Ponziani et al., 2019). This suggests that inflammation driven by gut microbiota may aggravate the progression of MASLD-HCC. This dysbiosis was confirmed by Behary et al. (2021) in MASLD-HCC and MASLD-cirrhosis patients. An increased rarity index was found in patients with MASH-HCC with cirrhosis (Sydor et al., 2020). Gut microbiota also function as cofactors in the pathogenesis of MASLD-HCC via interaction with immune cells. A recent study on 32 MASLD-HCC patients indicates that gut microbiota augment the expansion of IL-10+ Treg cells but reduce the expansion of CD8^+^ T cells (Behary et al., 2021). Gut microbiota dysbiosis contributes to the pathogenesis of MASLD-HCC in a spontaneous MASLD-HCC mouse model. Dietary cholesterol can trigger MASLD-HCC tumorigenesis by enhancing the abundance of *Desulfovibrio*, *Mucispirillum*, Desulfovibrionaceae, and *Anaerotruncus* and decreasing levels of *Bacteroides* and *Bifidobacterium* (Odenwald and Turner, 2017). Gut microbiota dysbiosis and alteration of gut bacterial metabolites contribute to the pathogenesis of MASLD-HCC in mice, and several probiotics including *Lactobacillus* and *Bifidobacterium* strains were identified as depleted in MASLD-HCC (Zhang X. et al., 2021).

### Role of lipotoxicity and glucotoxicity in MASH and HCC development

Hepatocytes function as a major site for fat accumulation, making them a main target of lipotoxicity (Rao et al., 2023; Yahoo et al., 2023). IR in adipose tissue results in enhanced release of free FAs and delivery to the liver, allowing for excessive lipid accumulation and toxic metabolite-induced lipotoxicity, causing mitochondrial dysfunction and endoplasmic reticulum stress (ERS) (Venkatesan et al., 2023; Yahoo et al., 2023). Increased free FA flux to the mitochondria enhances the rates of FAO, leading to increased ROS production (Serviddio et al., 2013). In MASLD, the damage in balance between antioxidant mechanisms and ROS production results in oxidative stress and thereby further induces mitochondrial dysfunction. Uncontrolled ROS production and the ensuing oxidative stress directly cause damage to cellular macromolecules such as DNA, proteins, and lipids (Chen et al., 2020). Lipotoxicity mediated by diacylglycerol and non-esterified FAs facilitates hepatic IR and ERS, which results in chronic inflammation, hepatic fibrosis, hepatic cirrhosis, and ultimately HCC (Samuel et al., 2010; Hardy et al., 2016; Piscaglia et al., 2016; Foerster et al., 2022). A recent study has revealed that decreased lysine-specific demethylase 6B (KDM6B), a key mediator of gene transcription, contributes to the development of MASLD-related HCC. This is accomplished by acquiring resistance to lipotoxicity via epigenetic downregulation of G0S2 expression-mediated activation of adipose triglyceride lipase/patatin-like phospholipase domain containing 2 (ATGL/PNPLA2) (Hatano et al., 2023). KDM6B loss may promote cell survival through activation of ATGL/PNPLA2 in NASH-related HCC. A high ATGL/PNPLA2 activation level was found in KDM6B-KO cells. Genetic or pharmacological inhibition of ATGL/PNPLA2 increased lipid accumulation and decreased cell proliferation in KDM6B-KO cells. ATGL/PNPLA2 overexpression facilitates the growth of HCC cells (Liu et al., 2019). Silencing G0S2 conferred lipotoxicity resistance in KDM6B-expressed HCC cells, whereas ATGL/PNPLA2 inhibition in the KDM6B-KO cells reduced these effects. These results indicate that targeting the KDM6B–G0S2–ATGL/PNPLA2 pathway may be a useful therapeutic strategy for MASLD-related HCC. Pharmacologically induced lipotoxicity comprising LXR agonists and Raf inhibitors represents a promising therapeutic strategy for the treatment of MASLD-related HCC (Rudalska et al., 2021).

## Current pharmacological therapies for MASH-driven HCC

**FIGURE 3:**
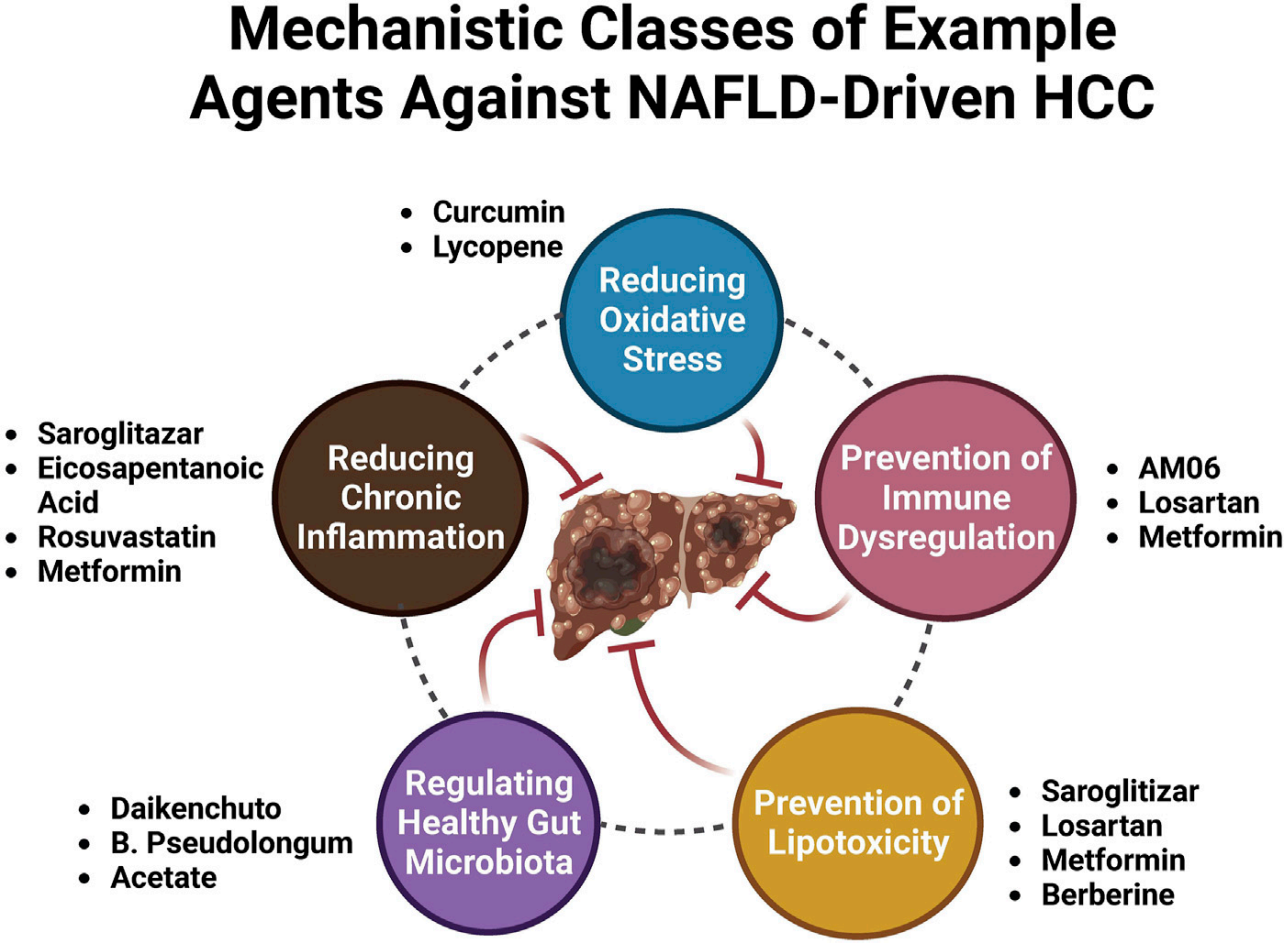
Mechanistic classes of example agents against MASLD-driven HCC.

Following a better understanding of the molecular mechanisms behind MASLD/MASH-HCC progression, research on anti-MASLD/MASH-HCC small molecules has intensified, resulting in an exponentially increasing number of published articles on this field. We present newly identified small molecules for the inhibition of MASLD-HCC disease progression (Table 1; Figure 3), describing drugs targeting anti-inflammatory, antioxidant, anti-fibrotic, anti-lipid metabolic, microbiota-pertaining, and immunomodulatory mechanisms.

**TABLE 1:** List of available potential compounds targeting different mechanisms to treat MASLD-driven HCC.

Compound	Experimental model	Involved mechanism	Effects	Ref
Saroglitazar	DEN + CDAHFD/C57BL/6 mice	Anti-inflammation	↓Liver injury markers (serum ALT and AST); ↓hepatic steatosis; ↓pro-inflammatory cytokines like TNF-α in the liver; ↑serum adiponectin and osteopontin levels; ↓hepatic tumors; ↓hepatic tumorigenesis	Giri et al. (2023)
9‐Xanthylacetic acid	CDAA HFD/C57BL/6J male mice	Anti-inflammation; anti-fibrosis	↓ Hepatic TAG accumulation; ↓ collagen α1 content; ↓ F4/80‐positive liver parenchyma; ↓ HCC development; ↓ body weight of mice; ↓ serum TGFα	Gnocchi et al. (2023)
Scoparone	HFD/DEN/C57BL/6J mice	Anti-inflammation	↓ Hepatic pathological changes; ↓ incidence and multiplicity of the tumor; ↑ liver functions; ↓ hepatic inflammation; ↓ NF-κB p65; ↓ TNF-α, MCP-1, iNOS, COX-2, NF-κB, and MMP-9; ↓ activation of the MAPK/Akt	Ye et al. (2023)
Tipifarnib	CDAHFD/DEN/C57BL/6J mice	Anti-inflammation	↓ HIF-1α; ↓ cell proliferation; ↑ apoptosis; ↓ IL-6; ↓ phosphorylated NF-κB; ↓ TGF-β; ↓ tumor nodule formation	Yamada et al. (2023a)
Eicosapentaenoic acid	DEN + HFD-fed mice	Anti-inflammation	↓ Hepatocarcinogenesis; ↓ activation of the pro-tumorigenic IL-6 effector STAT3	Inoue-Yamauchi et al. (2018)
Probiotics	Hepatocyte-specific PTEN knockout mouse	Anti-inflammation	↓ Serum transaminase levels; ↓ MASLD activity score; ↓ pro-inflammatory cytokines; ↓ liver fibrosis grade; ↓ the number of liver tumors; ↓ oxidative stress	Arai et al. (2022)
		Antioxidant		
Metformin	HFD-fed mice	Anti-inflammation	↓ Hepatocarcinogenesis; ↓ fat accumulation in the liver, associated with the suppression of adipose tissue inflammation	Tajima et al. (2013)
Apo-10′-lycopenoic acid	DEN + HFD-fed mice	Anti-inflammation	↓ Hepatic tumorigenesis and lung tumor incidence; ↑ hepatic SIRT1 protein and deacetylation of SIRT1 targets; ↓ caspase-1 activation and SIRT1 protein cleavage; ↑ glucose intolerance; ↓ hepatic inflammation	Ip et al. (2013)
Liraglutide	STZ + HFD-fed mice	Anti-inflammation	↓ Steatosis, inflammation, and hepatocyte ballooning; ↓ hepatocarcinogenesis	Kojima et al. (2020)
Vildagliptin	HFD-fed rat	Anti-inflammation	↓ Tumor progression by mediating the pro-angiogenic role of CCL2	Qin et al. (2018)
Rosuvastatin	HFD-fed mice	Anti-inflammation	↓ Hepatocarcinogenesis; ↓ TNF-α, IL-6, and TGF-β1; ↓ VEGFR, EGFR, and PDGF	Yokohama et al. (2016)
Lycopene	STZ + HFD-fed mice	Anti-inflammation	↓ Hepatocarcinogenesis; ↓ IL6; ↓ NF-kB p65 (Ser536) phosphorylation; ↓ STAT3 (Tyr705) phosphorylation	Ip et al. (2014)
Berberine	STZ + HFHC	Anti-inflammation; anti-angiogenesis	↓ Hepatocarcinogenesis; ↓ the expressions of genes related to lipogenesis, inflammation, fibrosis, and angiogenesis; ↓ phosphorylation of p38MAPK and ERK as well as COX2 expression	Luo et al. (2019)
Lycopene and tomato extract	DEN + HFD-fed rat	Antioxidant	↓ Hepatocarcinogenesis; ↓ glutathione S-transferase; ↓ cyclin D1; ↓ ERK; ↓ nuclear NF-κB; ↓ lipid peroxidation; ↓ cytochrome P450 2E1; ↓ inflammatory foci; ↓ mRNA expression of pro-inflammatory cytokines (TNF-α, IL-1β, and IL-12); ↑ nuclear Nrf2 and HO-1	Wang et al. (2010)
Curcumin	DEN + HFD-fed mice	Antioxidant	↓ Steatosis, fibrosis associated with decreasing serum aminotransferases; ↓ IFNγ, IL-1β, and IFNγ-inducible protein 10; ↓ VEGF, glypican-3, and prothrombin; ↑ Nrf2; ↓ hepatic C/EBPβ, CYP2E1, p-ERK1/2, and p67phox; ↓ SREBP1c	Afrin et al. (2017)
		Anti-inflammation		
		Anti-fibrosis		
		Inhibit lipogenesis		
NV556	STZ + HFD-fed mice	Anti-fibrosis	↓ The number and diameter of tumorous nodules; ↓ collagen deposition	Kuo et al. (2019)
Pioglitazone	DEN + CDAHFD-fed mice	Anti-fibrosis	↓ HCC development in both models; ↓ gross tumor nodules; ↓ fibrosis progression; ↓ activation of MAPK; ↑ AMPK	Li et al. (2019)
Ezetimibe	Pten ^Δhep^ mice were fed a HFD	Anti-fibrosis	↓ Tumor growth; ↓ cholesterol levels; ↓ angiogenetic processes; ↓ inflammation, liver fibrosis	Miura et al. (2019)
		Regulating lipid metabolism		
n-3 PUFAs	STZ/HFD-treated mice	Regulating lipid metabolism	↑ Survival of these mice; ↓ tumor size, and tumor number; ↑ hepatic n-3 PUFA content and n-3/n-6 PUFA ratio; ↓ hepatic lipid accumulation	Liebig et al. (2019)
Mulberry (*Morus alba* L.) leaf powder	STZ/HFD-treated mice	Regulating lipid metabolism	↓ Hepatocarcinogenesis; ↓ fat deposition	Wakame et al. (2022)
Daikenchuto (TU-100)	Spontaneous MASH TSOD mouse	Anti-inflammation	↓ The expression of IL6, IL1B, and ACTA2 mRNA in the liver; ↓ levels of serum alanine aminotransferase; ↓ MASH; ↓ tumor diameter; ↑ intestinal microbiome, the genera *Blautia and Ruminococcus*; ↓ Dorea and Erysipelotrichaceae	Yamada et al. (2023b)
		Regulating gut microbiota		
Curcumae	DEN + HFD-fed rat	Anti-inflammation	↓ IL-1β, IL-6, TNF-α, IL-2, and IL-7 in the serum and hepatic tissue; ↑ IL-10 in the serum and hepatic tissue; ↓ COX-2, PGE2, and NF-κB in the serum and hepatic tissue; ↑ gut microbial diversity and richness; ↓ abundance of genera *Mucispirillum* and *Clostridium*	Zhang et al. (2021b)
		Regulating gut microbiota		
Dietary tomato powder	DEN + HFD BCO1/BCO2 double knockout mice	Anti-inflammation	↓ HCC development (incidence, multiplicity, and tumor volume); ↓ hepatic inflammatory foci development; ↓ IL-1β, IL-6, IL-12α, monocyte chemoattractant protein-1, and iNOS (mRNA); ↑ sirtuin mRNA; ↑ hepatic circadian clock genes; ↑ gut microbial richness and diversity; ↓ relative abundance of the genera *Clostridium* and *Mucispirillum*	Xia et al. (2018)
		Regulating gut microbiota		
*B. pseudolongum*	DEN + HFHC-fed C57BL/6	Regulating gut microbiota	↓ MASLD-HCC formation in two mouse models; ↑ heathy gut microbiome composition; ↑ gut barrier function. Mechanistically, *B. pseudolongum*-produced acetate entered the portal vein to reach to the liver and bind to G coupled-protein receptor 43 (GPR43) on hepatocytes. GPR43 activation suppressed the IL-6/JAK1/STAT3 signaling pathway, thereby preventing MASLD-HCC progression	Song et al. (2023)
*B. pseudolongum*	MASLD-HCC cell lines (HKCI2 and HKCI10)	Regulating gut microbiota	MASLD-HCC cell co-incubation with B.p CM significantly suppressed cell proliferation, inhibited the G1/S phase transition, and induced apoptosis	Song et al. (2023)
Acetate	MASLD-HCC cell lines (HKCI2 and HKCI10)	Regulating gut microbiota	↓ Cell proliferation and induced cell apoptosis	Song et al. (2023)
Acetate	DEN + HFHC-fed C57BL/6	Regulating gut microbiota	↓ MASLD-HCC tumor formation *in vivo*	Song et al. (2023)
Mebendazole	Human MASLD-HCC cell lines (HKCI2 and HKCI10); MASLD-HCC xenografts	Inducing apoptosis	↓ MASLD-HCC growth; ↑ apoptosis; ↑ cellular senescence. Mebendazole synergized with navitoclax to inhibit MASLD-HCC cell growth via the induction of intrinsic and extrinsic apoptosis pathways	Yang et al. (2023b)
Metformin	Intrahepatic RIL-175 tumors of mice fed with MCD	Regulating immunity	Metformin treatment rescued the efficacy of anti-PD-1 therapy against liver tumors in MASH	Wabitsch et al. (2022)
AM06	STZ + HFD-fed mice	Regulating immunity	↓ MASH severity; ↓ progression of MASH to HCC; ↑ hepatic CXCR6+ natural killer T (NKT) cell; ↓ macrophage infiltration	Li et al. (2022)
Losartan	HFD/MUP-uPA mice	Regulating immunity	↓ Liver and peritumoral fibrosis; ↑ anti-PD-1-induced tumor regression; ↑ HCC infiltration by effector CD8^+^ T cells compared to the PD-1 blockade alone. The beneficial effects of losartan correlated with blunted TGF-β receptor signaling, reduced collagen deposition, and depletion of immunosuppressive fibroblasts	Gu et al. (2023)
Anti-CD122 antibody	High-fat/high-sucrose diet/C57BL/6 N	Regulating immunity	↓ Number of CXCR6+PD-1+ cells; ↑ OVA-specific CD8 activity; ↓ HCC growth compared to untreated MASH mice	Lacotte et al. (2023)
Metformin	HFD/transgenic zebrafish	Regulating immunity	↓ Macrophage polarization, liver size, and micronuclei formation in MASLD-associated HCC larvae; ↑ T-cell density in the liver, which was reversed by treatment with metformin	de Oliveira et al. (2019)
Honokiol	HFD-fed mice	—	↓ Hepatocarcinogenesis; ↓ EGFR signaling; ↑nuclear translocation of GR; ↑ MIG6/ERRFI1 expression, leading to EGFR degradation	Okuda et al. (2021)
Gallic acid	HFD-fed male C57BL/6J mice	—	MASLD-HCC progression	Zhang et al. (2023a)
T0901317–sorafenib	CD-HFD-fed male C57BL/6 mice mice	Inducing lipotoxicity	↓ MASH-induced tumor development	Rudalska et al. (2021)

AM06, breast milk-isolated *Akkermansia muciniphila*; AMPK, AMP-activated protein kinase; CCL2, chemokine ligand 2; CDAA, choline‐deficient L‐amino acid‐defined diet; CDAHFD, choline-deficient, L-amino acid-defined, high-fat diet; CD-HFD, choline-deficient high-fat diet; COX-2, cyclooxygenase; DEN, diethylnitrosamine; EGFR, epidermal growth factor receptor; ERRFI1, ERBB receptor feedback inhibitor 1; HFHC, high-fat and high-cholesterol diet; HFD, high-fat diet; FNγ, interferon γ; GR, glucocorticoid receptor; IL-1β, interleukin-1β; IL-6, interleukin-6; iNOS, inducible NO synthase; MAPK, mitogen-activated protein kinase; MCD, methionine/choline-deficient diet; MIG6, mitogen-inducible gene 6; NF-κB, nuclear factor kappa B; PDGF, platelet-derived growth factor; PGE2, prostaglandin E2; STZ, streptozotocin; TNF-α, tumor necrosis factor-α; VEGFR, vascular endothelial growth factor receptor.

### Anti-inflammatory drugs

Saroglitazar, a novel PPAR-α/γ agonist with predominant PPAR-α activity, has been shown to inhibit symptoms of MASH. A recent study has shown that it prevents the development of MASLD-HCC in rodents (Giri et al., 2023). Saroglitazar significantly reversed hepatic steatosis, reduced hepatic injury, and decreased the levels of pro-inflammatory cytokines in the livers of diethylnitrosamine (DEN)-treated, choline-deficient, L-amino acid-defined, high-fat diet (CDAHFD)-fed C57BL/6 mice. Saroglitazar completely prevented hepatic tumorigenesis (Giri et al., 2023). The compound 9-xanthylacetic acid (XAA) inhibits the development of MASLD-HCC (Gnocchi et al., 2023). The choline-deficient l-amino acid-defined diet (CDAAD) induced metabolic imbalance by stimulating lysophosphatidic acid receptor 6 (LPAR6) expression in mice. XAA reverses CDAAD-induced increase in hepatic lipid accumulation, inflammation, fibrosis, and HCC development. These findings are corroborated by the results of gain- and loss-of-function of LPAR6 in HCC cells (Gnocchi et al., 2023). Scoparone (SCO), a compound originating from the leaves and stems of *Artemisia capillaris*, has many pharmacological effects such as anti-tumor, lipid-lowering, anti-hypotensive, anti-inflammatory, analgesic, anti-coagulant, and anti-asthmatic (Li et al., 2021; Jiang et al., 2022; Wei et al., 2022; Wu X. et al., 2023; Shen et al., 2023; Zhou et al., 2023). The most recent study showed that scoparone attenuates the pathological alterations observed in MASLD-HCC mouse models. Scoparone inhibits activation of mitogen-activated protein kinase (MAPK)/Akt signaling and reverses upregulation of NF-κB p65 and its target genes, including NF-κB, TNF-α, cyclooxygenase (COX-2), MCP-1, iNOS, and MMP-9, in MASLD-HCC models (Ye et al., 2023). These results suggest that scoparone is a potential therapeutic agent for MASLD-HCC as it inhibits MAPK/Akt/NF-κB-mediated inflammatory pathways. Tipifarnib is a farnesyltransferase inhibitor (FTI) possessing anti-inflammatory and anti-tumor effects (Untch et al., 2018; Egawa et al., 2021; Shu et al., 2021; Greenberg et al., 2022; Smith et al., 2023). Tipifarnib significantly reduced tumor nodule formation and exhibited anti-tumor and anti-inflammatory effects in a MASH-related HCC mouse model challenged with DEN and a high-fat diet (HFD), primarily by decreasing serum IL-6 (Yamada et al., 2023). Tipifarnib strongly inhibited cell proliferation, decreased the expression of HIF-1α, and induced apoptosis. Tipifarnib suppressed IL-6 secretion *in vitro* and *in vivo* (Yamada et al., 2023). Additionally, tipifarnib suppressed the expression of phosphorylated NF-κB and TGF-β (Yamada et al., 2023). The omega-3 polyunsaturated fatty acid eicosapentaenoic acid (EPA) inhibits the development of HCC induced by DEN and HFD, suggesting that EPA attenuates the development of obesity-related MASLD-HCC by suppressing STAT3 (Inoue-Yamauchi et al., 2018). An increasing number of studies have shown that probiotics can prevent and mitigate the development of cancer (Deng et al., 2023; Zhao et al., 2023). Probiotics suppressed HCC pathogenesis by inhibiting the gene expression of pro-inflammatory cytokines and reducing oxidative stress in a hepatocyte-specific PTEN knockout mouse MASLD model (Arai et al., 2022). Metformin, a promising antidiabetic medication for cancer treatment, inhibits long-term HFD-induced HCC tumorigenesis by inhibiting liver fat accumulation in the early stage (before the onset of MASLD) in C57Bl/6 mice (Tajima et al., 2013; Saengboonmee et al., 2021; Wu et al., 2023; Georgopoulos et al., 2023). Apo-10′-lycopenoic acid (APO10LA), a cleavage metabolite of lycopene, significantly reduced hepatic tumorigenesis and lung metastasis in C57Bl/6J mice challenged with DEN and HFD. This was accomplished by increasing hepatic SIRT1 protein activity, deacetylating SIRT1 targets, decreasing caspase-1 activation, and reducing hepatic inflammation, indicating APO10LA as having anti-inflammatory and anti-tumor effects in MASLD-HCC models (Ip et al., 2013). Liraglutide, a glucagon-like peptide-1 receptor agonist used for the treatment of T2DM, obesity, and chronic weight management, completely suppressed hepatic tumorigenesis in mice with streptozotocin (STZ) and HFD-induced MASH models through ameliorating steatosis, inflammation, and hepatocyte ballooning (Kojima et al., 2020). The dipeptidyl peptidase 4 (DPP4) inhibitor vildagliptin prevented MASLD-HCC in mouse models challenged with DEN and HFD by reversing HFD-induced CCL-2 production and angiogenesis, suggesting that the DPP4/CCL2/angiogenesis axis plays a key role in inhibiting MASLD-HCC. Targeting DPP4 may represent a novel therapeutic regimen for MASLD-HCC (Qin et al., 2018). As a 3-hydroxy-3-methyl-glutaryl-coenzyme-A (HMG-CoA) reductase inhibitor, rosuvastatin inhibited hepatic tumorigenesis in an STAM mouse model challenged with a HFD by decreasing the expression of pro-inflammatory cytokines, suggesting that rosuvastatin may function as a preventive drug against MASLD-HCC (Yokohama et al., 2016). Lycopene suppresses hepatic tumorigenesis in HFD-promoted HCC by inactivating hepatic pro-inflammatory signaling and inflammatory foci (Ip et al., 2014). Berberine reduced the incidence of tumors and mitigated MASH in a STZ high-fat high-cholesterol diet (HFHC) mice model, suppressing lipogenesis, inflammation, fibrosis, and angiogenesis (Luo et al., 2019). Meanwhile, berberine inhibited the phosphorylation of p38 MAPK, extracellular signal-regulated kinase (ERK), and suppressed expression of COX2, suggesting berberine attenuates MASH-HCC by halting inflammation and angiogenesis via p38 MAPK/ERK-COX2 pathways (Luo et al., 2019).

### Antioxidant drugs

Dietary lycopene and tomato extract halt MASH-HCC in rats through antioxidant activity (Wang et al., 2010). Dietary lycopene from either tomato extract or pure compounds inhibited hepatic tumorigenesis in an MASLD-related HCC rat model challenged with DEN and a HFD. This was accomplished by inhibiting the activation of ERK and NF-κB, decreasing the expression of CYP2E1, reducing inflammatory foci, reducing pro-inflammatory cytokines, and increasing nuclear NF-E2-related factor-2 (Nrf2) and heme oxygenase-1 (HO-1) expression, suggesting that lycopene and tomato extract can inhibit MASH-HCC by reducing oxidative stress (Wang et al., 2010). Curcumin inhibits MASH-HCC pathogenesis in rats through antioxidant activity (Afrin et al., 2017). Curcumin inhibited hepatic tumorigenesis in a C57BL/6J male mice MASLD-related HCC model challenged with STZ and a HFD by inhibiting hepatic C/EBPβ, CYP2E1, p-ERK1/2, and p67phox, while upregulating Nrf2 (Afrin et al., 2017). Curcumin significantly reduced the translocation of high-mobility group box 1 (HMGB1) into the cytosol and decreased the protein expression of toll-like receptor 4 (TLR4). Curcumin reduced MASH-HCC by downregulating the protein expression of glypican-3, prothrombin, and vascular endothelial growth factor (VEGF) (Afrin et al., 2017).

### Anti-fibrosis drugs

The cyclophilin inhibitor derived from sanglifehrins NV556 reduces MASH-HCC by inhibiting fibrosis (Kuo et al., 2019). NV556 inhibited hepatic tumorigenesis in a mice model challenged with STZ and a HFD by inhibiting fibrosis, rather than altering inflammation, steatosis, and systemic cytokine generation, suggesting NV556 as a promising agent for the treatment of MASH-driven fibrosis and HCC (Kuo et al., 2019). Pioglitazone effectively reduced hepatic tumorigenesis in an MASH-related HCC mice model challenged with DEN and choline-deficient, L-amino acid-defined, high-fat diet (CDAHFD) by inhibiting fibrosis via inactivating MAPK and upregulating the hepatoprotective AMP-activated protein kinase (AMPK) pathway (Li et al., 2019). An inhibitor of cholesterol absorption, ezetimibe, reduces MASH-HCC by inhibiting fibrosis (Miura et al., 2019). Ezetimibe inhibited hepatic tumorigenesis in hepatocyte-specific phosphatase and tensin homolog (Pten)-deficient (Pten^Δhep^) mice challenged with a HFD by suppressing liver fibrosis and inflammation (Miura et al., 2019).

### Drugs regulating lipid metabolism

Dietary n-3 polyunsaturated fatty acids (PUFAs) reduced hepatic tumorigenesis in an MASLD-related HCC mice model challenged with STZ and a HFD by regulating lipid metabolism, evidenced by decreased hepatic lipid accumulation and increased hepatic content of n-3 PUFAs and a higher n-3/n-6 PUFA ratio, suggesting n-3 PUFAs as a new therapeutic regimen for MASLD-HCC (Liebig et al., 2019). Dietary mulberry (*Morus alba* L.) leaf powder reduced hepatic tumorigenesis in an MASLD-related HCC C57L/6J mice model challenged with STZ and a HFD by regulating lipid metabolism, evidenced by reduced fat deposition and adenoma. This study suggests that the administration of mulberry leaf powder in STAM mice inhibits the progression of MASH-HCC, highlighting that it may be effective in preventing the development of MASH-HCC in humans (Wakame et al., 2022).

### Regulating gut microbiota

Daikenchuto (TU-100) reduced hepatic tumorigenesis in Tsumura–Suzuki obese diabetes mice with the spontaneous onset of MASH and HCC by modulating the intestinal microbiome, evidenced by increased *Blautia and Ruminococcus* genera and decreased *Dorea and Erysipelotrichaceae* genera (Yamada et al., 2023). Curcumae reduced hepatic tumorigenesis in an MASLD-related HCC mice model challenged with DEN and a HFD by suppressing the levels of pro-inflammatory cytokines and inflammatory mediators including prostaglandin E2 (PGE2), COX-2, and NF-κB and additionally augmented the level of IL-10 in the hepatic tissue and serum. Furthermore, Curcumae enhanced the diversity and richness of gut microbiota and decreased the relative abundance of *Clostridium* and *Mucispirillum* (Zhang et al., 2021). This study suggests that Curcumae attenuates MASLD-HCC via regulating gut microbiota, along with inhibiting oxidative stress and its associated inflammation. Dietary tomato powder inhibits MASLD-HCC by regulating gut microbiota in mice with loss of carotenoid cleavage enzymes (Xia et al., 2018). Dietary tomato powder reduced hepatic tumorigenesis in a β-carotene-15, 15′-oxygenase (BCO1)/BCO2 double-knockout mice model challenged with DEN and HFD. This was accomplished by decreasing hepatic inflammatory foci development and expression of pro-inflammatory genes, increasing the expression of SIRT1, hepatic circadian clock genes, and nicotinamide phosphoribosyltransferases (Xia et al., 2018). Dietary tomato powder also increased gut microbial diversity and richness, significantly decreasing the relative abundance of the genera *Clostridium* and *Mucispirillum* (Xia et al., 2018). This study indicates that dietary tomato powder prevents MASLD-HCC by inhibiting inflammation and modulating gut microbiota independent of carotenoid cleavage enzymes (Xia et al., 2018). Recent studies have shown that *Bifidobacterium pseudolongum* is a potential novel probiotic for the prevention of MASLD-HCC. Mechanistically, it was found to suppress MASLD-HCC progression by secreting acetate, which by binding to the hepatic G-coupled-protein receptor 43 (GPR43) suppresses the activation of hepatic oncogenic IL-6/JAK1/STAT3 signaling pathways (Song et al., 2023).

### Regulating immunity

MASH impaired the effect of anti-PD-1 therapy by inducing a pro-inflammatory phenotypic change and impairing the metabolism of hepatic CD8^+^ T cells in multiple murine MASH liver cancer models. Reduced motility of intratumoral CD8^+^ T cells was found through *in vivo* imaging analysis. Metformin was found to modulate the anti-PD-1 therapy efficacy against liver tumors in MASH models (Wabitsch et al., 2022). HFD alters macrophage polarization and promotes the liver inflammatory microenvironment, exacerbating cancer progression in MASLD/MASH-associated zebrafish HCC models. Metformin reversed MASLD/MASH-HCC by modulating the immune response via altering T-cell infiltration and macrophage polarization (de Oliveira et al., 2019). Gut *Akkermansia muciniphila* is reduced in mice and patients with MASH-related HCC (Li et al., 2022). Breast milk-isolated *A. muciniphila* (AM06) improved the severity of MASH, in addition to inhibiting the pathogenesis of MASH-HCC, indicated by decreased macrophage infiltration and an increased level of hepatic CXCR6^+^ natural killer T (NKT) cells (Li et al., 2022). The anti-tumor effects of *A. muciniphila* were attenuated by NKT cell deficiency in mice (CD1d^−/−^ and CXCR6^−/−^). *A. muciniphila* enhanced the NKT cell-mediated killing of HepG2 cells (Li et al., 2022). Peritumoral fibrosis exerts an obstacle to T-cell-mediated tumor regression in mouse models of MASH-HCC. The antihypertensive drug angiotensin II receptor inhibitor losartan inhibited liver and peritumoral fibrosis, thereby substantially boosting tumor regression induced by anti-PD-1 therapy, mostly by facilitating effector CD8^+^ T-cell-mediated infiltration. The beneficial effects of losartan are associated with blunting of TGF-β receptor signaling, reduced fibrosis, and depletion of immunosuppressive fibroblasts (Gu et al., 2023).

## Conclusion and future perspectives

This review summarizes the recent progress of research on the pathological pathways and underlying mechanisms of MASLD/MASH-HCC and reviews the application of compounds in the treatment of MASLD-HCC. This review is expected to improve our knowledge of the molecular mechanisms of MASLD/MASH-HCC and highlight strategies for targeting MASLD/MASH-HCC by pharmacological modulation as potential novel therapeutic targets. The progression of MASLD/MASH to HCC is still an international research hotspot, and there has been some progress in elucidating metabolic disorder/gut microbiota imbalance and immune factors in the progression of MASLD/MASH to HCC. However, the exploration of treatment for MASLD/MASH-HCC is still in its infancy. In this article, based on a brief review of the latest research on the pathogenesis and progress of MASLD-HCC, we further summarize and organize the latest results of compounds in the treatment of MASLD-HCC. Based on the main mechanisms of anti-inflammatory, antioxidant, gut microbiota regulation, lipid metabolism regulation, and liver fibrosis inhibition, we classified the drugs used to treat MASLD/MASH-HCC and elucidated their pharmacological effects and related mechanisms of action. Adding further complexity, the same drug may inhibit MASLD/MASH-HCC through one or more of the above mechanisms, adding new insights for future polyreceptor pharmacology. Although continuous exploratory experimental research is still ongoing, the exact molecular mechanisms of these drugs in treating MASLD/MASH-HCC still need to be further explored.

After our detailed discussion, it is important to recall limitations that still plague the field of MASLD/MASH-HCC research. The first limitation is that the etiology of MASLD/MASH-HCC remains largely unclear, and further research is still needed. Recent studies have shown that new mechanisms of regulated cell death, such as ferroptosis, can be involved in the development and progression of MASLD (Feng et al., 2022; Wang et al., 2022; Zhang et al., 2023b; Zou et al., 2023) to HCC (Cong et al., 2022; Wang et al., 2023a; Wang et al., 2023b; Xu et al., 2023), and modulating ferroptosis may be a potential novel therapeutic target for MASLD and HCC (Yin et al., 2022; Ajoolabady et al., 2023; Cheng et al., 2023). However, mechanistic details relating to ferroptosis and the molecular pathways concerning transition of MASLD to HCC have been lacking. It is of fundamental importance to understand the importance of ferroptosis in the pathogenesis of MASLD-HCC. The second limitation is that the mechanism of specific genetic factors in the progression of MASLD/MASH to HCC and the exact downstream molecules are currently unclear, and further research is needed. The third limitation is that there is a lack of large-scale clinical trials on targeted MASLD/MASH-HCC drugs. The fourth limitation is that it is still largely unclear how one may develop or repurpose existing drugs for new clinical indications.

Due to a significant increase in obesity and T2DM globally, MASLD has gradually become an important cause of HCC, leading to the occurrence of MASLD-HCC. To note, HCC is characterized by high heterogeneity and different genetic mutations that could damage the effectiveness of current treatments. Therefore, future clinical trials should consider the most representative genetic mutations and find new strategies to identify biomarkers to guide personalized and precision treatment for HCC. Currently, there are no approved effective drugs to treat MASLD/MASH, implying that the targeted inhibition of MASLD/MASH and preventing disease progression to HCC have important social significance and are worthy of further exploration.
